# The effects of physical activity and platelet-high-density lipoprotein cholesterol ratio on the risk of anxiety disorders: A cross-sectional study based on NHANES 2015–2023

**DOI:** 10.1371/journal.pone.0335122

**Published:** 2025-10-27

**Authors:** Liu Jiahui, Zhou Yifan, Lan Yi, Zhang Weihao, Yuan Dian, Jian Sun

**Affiliations:** 1 Wushu College, Wuhan Sports University, Wuhan, Hubei, China; 2 Northeast China Ethnic Traditional Sports Research Center, Wuhan Sports University, Wuhan, Hubei, China; Ankara University: Ankara Universitesi, TÜRKIYE

## Abstract

Anxiety disorders are a global mental health issue that is strongly associated with lifestyle factors. Physical activity (PA) could benefit anxiety symptoms, though the precise effect remains controversial. The platelet-to-high-density lipoprotein cholesterol (HDL-C) ratio (PHR) is an emerging biomarker and has gained attention for its link to mental health. This study used data from the 2015−2023 National Health and Nutrition Examination Survey (NHANES) to examine the effects of PA and PHR on the risk of anxiety disorders. NHANES 2015−2023 data were used to examine participants’ PA, PHR, and their relationship to anxiety symptoms using a cross-sectional design. Moreover, logistic regression, subgroup, and unrestricted cubic spline analyses were employed in this study. The analysis results indicated that higher intensity PA significantly reduced the risk of anxiety disorders (OR = 0.824, 95% CI: 0.716–0.948, *p* < 0.05). Both moderate PHR (OR = 0.769, 95% CI: 0.664–0.891, *p* < 0.001) and higher PHR (OR = 0.781, 95% CI: 0.670–0.911, *p* < 0.05) were significantly associated with reduced anxiety risk compared to the lowest quartile of PHR. A non-linear relationship was observed, and the risk was reduced when PHR ranged between 4.51 and 10.18, and increased when PHR was outside this range. Subgroup analysis results revealed significant variations in effects across age, gender, lifestyle, and chronic disease groups. This study confirmed the significant role of PA and PHR in regulating anxiety disorder risk. Future interventions should tailor PA intensity and PHR levels to individual differences to improve the prevention and treatment of anxiety disorders.

## Introduction

Cardiovascular disease (CVD) represents a major global public health challenge and has been the leading cause of global death for many years [1]. According to the World Health Organization (WHO), the morbidity and mortality of CVD are increasing in high- and middle-income countries, with the increasingly growing burden, especially in aging societies [2]. Concurrently, anxiety disorders, as a widespread mental health issue, significantly affect the quality of life of people worldwide [3]. Anxiety disorders not only affect mental health but are also closely linked to the development of various physiological diseases, especially CVD [4]. Chronic anxiety triggers psychological stress, resulting in various physiological responses (such as elevated blood pressure, increased heart rate, and impaired immune function), which, in turn, increase the risk of CVD [5]. In recent years, the changes in modern lifestyles and the increase in sedentary behavior have brought the interaction between mental and cardiovascular health to the forefront of research. It has been suggested that physical activity (PA) plays a crucial role in moderating the relationship between anxiety disorders and CVD [6]. Despite its significant mental health benefits, PA also improves cardiovascular health [7]. However, the physiological mechanisms by which PA moderates the risk of anxiety disorders, especially by influencing physiological markers in the blood such as the platelet-to-high-density lipoprotein cholesterol (HDL-C) ratio (PHR), remain insufficiently explored.

PHR is increasingly recognized as a novel biomarker for the assessment of CVD risk [7]. It has been shown that platelet overactivation is strongly associated with cardiovascular events [8]. However, HDL-C exerts anti-atherosclerotic and anti-thrombotic effects, playing a key role in protecting cardiovascular health [9]. PHR values have significant implications for both cardiovascular and mental health by integrating the risk of thrombosis [10]. Regular PA has been shown to effectively improve blood lipid levels, particularly by increasing HDL-C and reducing platelet activity, thereby optimizing PHR values and exerting protective effects on cardiovascular health [11]. More importantly, PA may indirectly alleviate anxiety symptoms through the following physiological mechanisms. Specifically, anxiety disorders are frequently associated with an overactive sympathetic nervous system, which may lead to abnormal platelet function and reduced HDL-C levels; these physiological changes could exacerbate anxiety symptoms and increase cardiovascular risk [12].

Therefore, PA not only improves lipid metabolism and platelet activity but may also reduce anxiety symptoms and lower cardiovascular risk by optimizing PHR values [13]. Furthermore, PA significantly improves anxiety symptoms by promoting neurotransmitter homeostasis and reducing physiological stress. Regular exercise not only improves self-efficacy but also effectively alleviates anxiety by reducing inflammatory responses and elevating mood [14]. However, the existing research lacks sufficient evidence, especially in the form of validation using large population samples, and cannot systematically explore how PA affects the risk of anxiety disorders through this physiological mechanism.

This study aimed to explore the association between PA, PHR, and the risk of anxiety disorders using data from the National Health and Nutrition Examination Survey (NHANES). Through cross-sectional data analyses, this study assessed how PA alleviated the risk of anxiety disorders by improving PHR. The findings of this study may provide an empirical basis for clinical intervention and scientific support for developing more effective prevention strategies for anxiety disorders. Additionally, this study explored how PA can bridge the gap between cardiovascular and mental health by improving blood physiological markers, particularly PHR, providing a theoretical basis for future public health policies and mental health interventions.

## Methods

### Data collection

**Fig 1:**
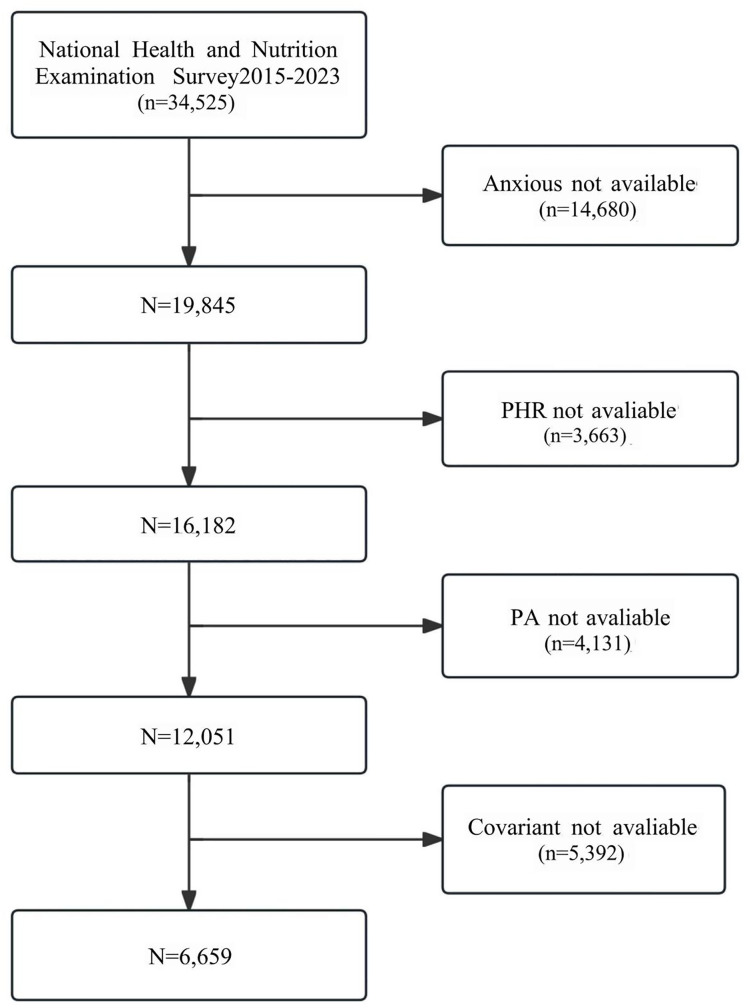
Flowchart of the sample selection from NHANES (2015–2023).

Data for this study were obtained from the NHANES, a nationally representative health survey sponsored by the Centers for Disease Control and Prevention (CDC) that assesses the health, nutritional status, and disease burden in adults and children in the United States. NHANES data from 2015 to 2023 were selected for this study because this period encompasses a wealth of health behaviors, mental health assessments, and biomarker information, providing a valuable reference for studying the association between PA and anxiety disorders. With this nationally representative dataset, the present study can further explore the relationship between PA, blood biomarkers, and anxiety disorders, advancing the scientific exploration of the potential association between mental health and biomarkers. The exclusion criteria for the subjects were: (1) individuals under 20 years of age; (2) participants lacking information on anxiety status; (3) samples with unreliable data quality; and (4) individuals who did not provide data on PA or PHR. The selection process for the study sample is illustrated in Fig 1.

### Ethical approval and informed consent

All study participants provided written informed consent, and the study design was approved by the Ethics Review Board of the National Center for Health Statistics. Since NHANES data are publicly available and accessible via the internet, no additional application for medical ethics committee approval was required.

### Variables

#### PA assessment.

The Global Physical Activity Questionnaire (GPAQ) was applied for data collection in this study. The GPAQ was developed by the WHO to assess PA levels across leisure, occupational, and transport-related activities [15]. PA was converted into Metabolic Equivalent (MET) minutes per week for moderate to vigorous activity, based on the WHO analytical guidelines [16]. Different types of exercise correspond to different MET values, and NHANES provides recommended MET values for each activity type. MET values for PA were determined based on the type of activity, weekly frequency, and duration, and were calculated using the following formula: PA (MET-min/wk) = MET * weekly frequency * duration for each activity type [17]. Furthermore, based on the weekly MET-min/wk value, PA intensity was categorized into mild intensity (600–3000 MET-min/wk), low intensity (below 600 MET-min/wk), and high intensity (above 3000 MET-min/wk) [18].

#### PHR assessment.

In this study, the PHR was calculated as follows [19]:


PHR=platelet count(thousands/μL)÷HDL−C concentration(mg/dL).

Platelet counts and HDL-C concentrations were obtained through standard blood test methods following the criteria provided by NHANES data. Specifically, platelet counts were determined by a complete blood count (Automated Hematology Analyzer), and the results were reported in thousands/µL. On the other hand, HDL-C concentration was measured by standardized lipid assays [e.g., enzyme-linked immunosorbent assay (ELISA) or chemiluminescent assay], and the results were expressed in mg/dL. Based on the analysis results, PHR values were divided into four quartile categories: first quartile (Q1; PHR values in the lowest 25^th^ percentile); second quartile (Q2; PHR values between the 25^th^ and 50^th^ percentiles); third quartile (Q3; PHR values between the 50^th^ and 75^th^ percentiles); and fourth quartile (Q4; PHR values in the highest 25^th^ percentile).

#### Anxious assessment.

In this study, anxiety was assessed through the Self-Rating Anxiety Scale (SAS) during individual interviews. Specifically, the respondents were asked: “How often do you feel worried, nervous, or anxious?” This question was used to assess the frequency of anxiety symptoms. The presence or absence of an anxiety state could be evaluated based on the respondent’s description of the frequency of anxiety experiences. Specifically, based on the respondents’ answers, anxiety states were categorized into two groups: without anxiety [No; respondents reported feeling anxious only occasionally in the past year or never feeling worried, nervous, or anxious (e.g., several times a year or never] and with anxiety (Yes; respondents reported frequent feelings of anxiety in the past year, with symptoms occurring on a daily, weekly, or monthly basis, and these symptoms affected their daily life.

#### Covariate assessment.

Based on previous research and recommendations from clinical experts, several potential confounding and modifying variables were identified that may influence the relationship between PA, PHR, and the risk of anxiety disorders. Specifically, these variables included: age, gender (male or female), race (non-Hispanic white, non-Hispanic black, Mexican American, and others), education level (from less than 9th grade to college or above), marital status (e.g., married, widowed, divorced, etc.), poverty-to-income ratio (PIR, household income classified as poor, low-moderate, upper-moderate, or high-income), body mass index (BMI, calculated as weight in kilograms divided by the square of height in meters), smoking status, drinking habits, sleep duration, and the presence or absence of chronic diseases (e.g., diabetes, high blood pressure, heart disease, etc.) [20]. These variables were used to control for potential confounding effects, ensuring the accuracy and comprehensiveness of the study results.

### Statistical analysis

Data in this study were analyzed using SPSS (27.0) and R (4.4.1). First, the distribution of all variables was assessed through descriptive statistics, including the mean, standard deviation (SD), frequency, and percentage. The mean ± SD was employed for continuous variables, while frequencies and percentages were applied to categorical variables.

Univariate analyses were performed to assess the relationship between each latent variable and the risk of anxiety disorders. The association between categorical variables and anxiety disorders was examined using the chi-square test (χ²), while independent samples *t*-tests or one-way analyses of variance (ANOVA) were applied to compare differences in continuous variables across groups with different anxiety states [21].

Multivariate analyses were conducted to further explore the relationship between PA, PHR, and the risk of anxiety disorders while controlling for potential confounding variables. Variables (including age, gender, race, education level, marital status, income level, BMI, smoking status, sleep duration, and the presence of chronic diseases) were included in the model. Specifically, in the multivariate regression model, anxiety status (with/without anxiety) was the dependent variable, while PA level and PHR were the primary independent variables.

Moreover, to further explore the relationship between PA, PHR, and anxiety, three logistic regression models were developed to examine their correlation. In model I, no adjustments were made for confounders. In model II, adjustments were made for age, gender, and BMI to control for potential factors that may influence anxiety. Model III was established based on model II by adjusting for education level, marital status, household PIR, diabetes mellitus, hypertension, coronary heart disease, and sleep duration [22].

Subsequently, subgroup analyses were conducted to explore the potential impact of demographic characteristics, lifestyle, and chronic disease status on the research results. The analyses considered multiple factors (such as age, gender, BMI, education level, marital status, and the presence of chronic diseases), aiming to reveal the differences in the relationship between PA and the risk of anxiety disorders across various groups. Additionally, the current study explored how lifestyle factors (including sleep quality and smoking behavior) influence the relationship between PA and anxiety disorders.

Furthermore, interaction effect analyses were conducted to test the potential interaction between PA and PHR on the risk of anxiety disorders, assessing the joint effect of these two factors by incorporating interaction terms.

Statistical significance for all analyses was set at *p* < 0.05 with two-sided tests. Data were analyzed using SPSS (27.0) for basic statistical and regression analyses, and R (4.4.1) for more complex model construction and interaction effect analyses.

## Results

### Baseline characteristics

A total of 34,525 respondents participated in this study from 2015 to 2023, of whom 19,845 provided complete data on anxiety symptoms. However, 13,220 participants were further excluded due to missing data on PA, PHR, and other covariates. Eventually, 6,625 respondents were included in this analysis, with a mean age of 48.87 ± 17.06 years, and 50.9% were male. In the anxiety assessment, 52.2% of the respondents were classified as “anxious” and 47.8% as “not anxious.” Additionally, the distribution of PA indicated that 33.8% (n = 2,236) of the respondents engaged in high-intensity activities weekly, 43.8% (n = 2,901) in mild-intensity activities, and 22.5% (n = 1,488) in low-intensity activities. The respondents enrolled in this analysis were diverse in terms of age, gender, race, education level, marital status, PIR, BMI, smoking status, sleep duration, and chronic disease status. Detailed characteristics of the participants are presented in Table 1.

**Table 1:** Baseline characteristics of participants with anxiety disorders.

Characteristics	PA Status				P
	Total (n = 6625)	High (n = 2236,33.8%)	Mild (n = 2901,43.8%)	Low (n = 1488,22.5%)	
**Age, year** **(mean, se)**	48.47 ± 17.06	44.53 ± 16.66	49.78 ± 17.03	51.41 ± 16.69	<0.001
**BMI, kg/m2** **(mean, se)**	29.56 ± 7.14	29.31 ± 6.88	29.42 ± 6.90	30.15 ± 7.70	0.024
**Family PIR** **(mean, se)**	2.99 ± 1.63	2.73 ± 1.61	3.16 ± 1.60	3.10 ± 1.64	0.003
**Sleep duration** **(mean, se)**	7.60 ± 1.46	7.57 ± 1.47	7.67 ± 1.38	7.68 ± 1.51	<0.001
**Gender (n, %)**	<0.001				
**Male**	3370 (50.9%)	1349 (60.3%)	1334 (46.0%)	687 (46.2%)	
**Female**	3255 (49.1%)	887 (39.7%)	1567 (54.0%)	801 (53.8%)	
**Race (n, %)**	<0.001				
**Mexican American**	741 (11.2%)	326 (14.6%)	270 (9.3%)	145 (9.7%)	
**Other Hispanic**	630 (9.5%)	239 (10.7%)	269 (9.3%)	122 (8.2%)	
**Non-Hispanic White**	3254 (49.1%)	957 (42.8%)	1510 (52.1%)	787 (52.9%)	
**Non-Hispanic Black**	1084 (16.4%)	434 (19.4%)	414 (14.3%)	236 (15.9%)	
**Non-Hispanic Asian**	545 (8.2%)	153 (6.8%)	283 (9.7%)	109 (7.3%)	
**Other Race – Including Multi-Racial**	371 (5.6%)	127 (5.7%)	155 (5.3%)	89 (6.0%)	
**Education level (n, %)**	<0.001				
**Less than 9th grade**	231 (3.5%)	98 (4.4%)	86 (3.0%)	47 (3.1%)	
**9-11th grade (Includes 12th grade with no diploma)**	489 (7.4%)	228 (10.2%)	157 (5.4%)	104 (7.0%)	
**High school graduate/GED or equivalent**	1349 (20.4%)	547 (24.4%)	506 (17.4%)	296 (19.9%)	
**Some college or AA degree**	2216 (33.4%)	813 (36.4%)	930 (32.0%)	473 (31.8%)	
**College graduate or above**	2340 (35.3%)	550 (24.6%)	1222 (42.1%)	568 (48.2%)	
**Marital status (n, %)**	0.733				
**Married**	3514 (53.0%)	1095 (49.0%)	1593 (54.9%)	826 (55.5%)	
**Widowed**	740 (11.2%)	138 (6.2%)	372 (12.8%)	230 (15.5%)	
**Divorced**	941 (14.2%)	252 (11.3%)	437 (15.1%)	252 (16.9%)	
**Separated**	123 (1.9%)	54 (2.4%)	38 (1.3%)	31 (2.1%)	
**Never married**	848 (12.8%)	424 (19.0%)	323 (11.2%)	101 (6.8%)	
**Living with partner**	459 (6.9%)	273 (12.2%)	138 (4.8%)	48 (3.2%)	
**Diabetes (n, %)**	0.640				
**YES**	717 (10.8%)	177 (7.9%)	337 (11.6%)	203 (13.6%)	
**NO**	5730 (86.5%)	2017 (90.2%)	2473 (85.2%)	1240 (83.4%)	
**Borderline**	178 (2.7%)	42 (1.9%)	91 (3.1%)	45 (3.0%)	
**High blood pressure (n, %)**	<0.001				
**YES**	2144 (32.4%)	636 (28.5%)	950 (32.8%)	558 (37.5%)	
**NO**	4481 (67.6%)	1600 (71.5%)	1951 (67.2%)	930 (62.5%)	
**Heart disease (n, %)**	0.002				
**YES**	238 (3.6%)	53 (2.4%)	126 (4.3%)	59 (4.0%)	
**NO**	6387 (96.4%)	2183 (97.6%)	2775 (95.7%)	1429 (96.0%)	
**Smoking (n, %)**	<0.001				
**YES**	2918 (44.0%)	1087 (48.6%)	1205 (41.6%)	626 (42.1%)	
**NO**	3707 (56.0%)	1149 (51.4%)	1696 (58.4%)	862 (57.9%)	
**Alcohol consumption (n, %)**	0.066				
**YES**	4340 (65.5%)	1499 (67.0%)	1883 (65.0%)	958 (64.4%)	
**NO**	2285 (34.5%)	737 (33.0%)	1018 (35.0%)	530 (35.6%)	
**PHR (n, %)**	0.002				
**Class1**	1656 (25.0%)	589 (26.4%)	693 (23.9%)	374 (25.1%)	
**Class2**	1661 (25.1%)	528 (23.6%)	742 (25.6%)	391 (26.3%)	
**Class3**	1648 (24.9%)	544 (24.3%)	731 (25.2%)	373 (25.1%)	
**Class4**	1660 (25.1%)	575 (25.7%)	735 (25.3%)	350 (23.5%)	
**Anxious (n, %)**	<0.001				
**YES**	3460 (52.2%)	1122 (50.2%)	1552 (53.5%)	786 (52.8%)	
**NO**	3165 (47.8%)	1114 (49.8%)	1349 (46.5%)	702 (47.2%)	

### Logistic regression

The results of the three weighted logistic regression models are listed in Table 2. Model 1 was unadjusted; model 2 was adjusted for gender, age, and BMI; and model 3 was adjusted for all covariates. After adjustment for multiple confounders, the associations between PA levels, PHR, and anxiety disorders remained significant.

**Table 2:** The association of PA and PHR with anxiety disorders.

Exposure	OR (95%CI), P-value		
	Model1	Model2	Model3
**PA status**			
High	0.895 (0.785, 1.021) 0.099	0.832 (0.725, 0.954) 0.008	0.824 (0.716, 0.948) 0.007
Mild	1.024 (0.904, 1.161) 0.708	0.977 (0.860, 1.111) 0.726	0.968 (0.850, 1.102) 0.625
Low	[Reference]	[Reference]	[Reference]
P for trend	0.049	0.008	0.008
**PHR classification**			
1	0.858 (0.748,0.983) 0.028	0.785 (0.675, 0.912) 0.002	0.781 (0.670, 0.911) 0.002
2	0.809 (0.705,0.927) 0.002	0.765 (0.662, 0.883) <0.001	0.769 (0.664, 0.891) <0.001
3	0.920 (0.802,1.055) 0.232	0.878 (0.762, 1.011) 0.071	0.886 (0.768, 1.023) 0.098
4	[Reference]	[Reference]	[Reference]
P for trend	0.016	0.001	0.002

#### PA levels and anxiety disorders.

According to multivariate regression analysis results, higher levels of PA significantly reduced the risk of anxiety disorders. High-intensity PA was associated with a significant reduction in anxiety disorder risk in Model 3 (OR: 0.824, 95% CI: 0.716–0.948, *p* = 0.007) compared to low-intensity PA (control group). However, mild-intensity PA did not show a significant change in anxiety disorder risk in any of the models (*p* > 0.05). The trend test results revealed a significant negative association between PA levels and the risk of anxiety disorders (*p* for trend = 0.008).

#### Relationship between PHR classification and anxiety disorders.

As indicated by the PHR classification analysis results, higher PHR values (Classification 1 and Classification 2) significantly reduced the risk of anxiety disorders compared to the lowest PHR values (controls). Specifically, the results for Classification 1 were significant in model 3 (OR: 0.781, 95% CI: 0.670–0.911, *p* = 0.002), and the results for Classification 2 exhibited a similar significant trend in model 3 (OR: 0.769, 95% CI: 0.664–0.891, *p* < 0.001). However, the results for Classification 3 in model 3 did not show statistical significance (*p* > 0.05). Additionally, the trend test results revealed a significant negative association between PHR and the risk of anxiety disorders (*p* for trend = 0.002).

### Unrestricted cubic splines

**Fig 2:**
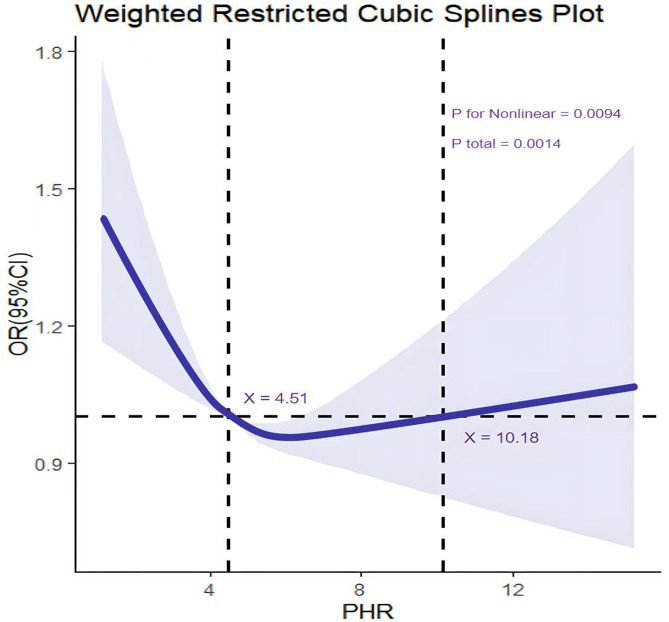
Weighted restricted cubic spline plot.

The nonlinear association between PA and PHR with the risk of anxiety disorders was explored using unrestricted spline analysis, and the results are presented in Fig 2. The analysis results revealed a significant nonlinear association between PA and the risk of anxiety disorders (*p* for nonlinear = 0.0241), but the overall association did not reach a statistical significance (*p* total = 0.0729). In contrast, the nonlinear association between PHR and the risk of anxiety disorders was statistically significant (*p* for nonlinear = 0.0094). The risk (OR value) for the target variable was the lowest when the PHR value was approximately 4.51, suggesting that a PHR value within this range may represent the optimal level for minimizing the risk. However, as the PHR value further increased (PHR > 10.18), the OR value gradually increased, suggesting that either very high or very low PHR values may elevate the risk of the target variable (Fig 2).

### Subgroup analysis

The relationship between PA, PHR, and anxiety symptoms was further explored using subgroup analysis. The results revealed significant differences in their association with anxiety symptoms across groups with varying demographic characteristics. These differences suggested that multiple factors (such as age, BMI, marital status, chronic disease status, and lifestyle) may play significant moderating roles in the effects of PA and PHR on anxiety. The results of the subgroup analysis on PA and PHR are presented below.

#### Association between PA and anxiety symptoms.

**Fig 3:**
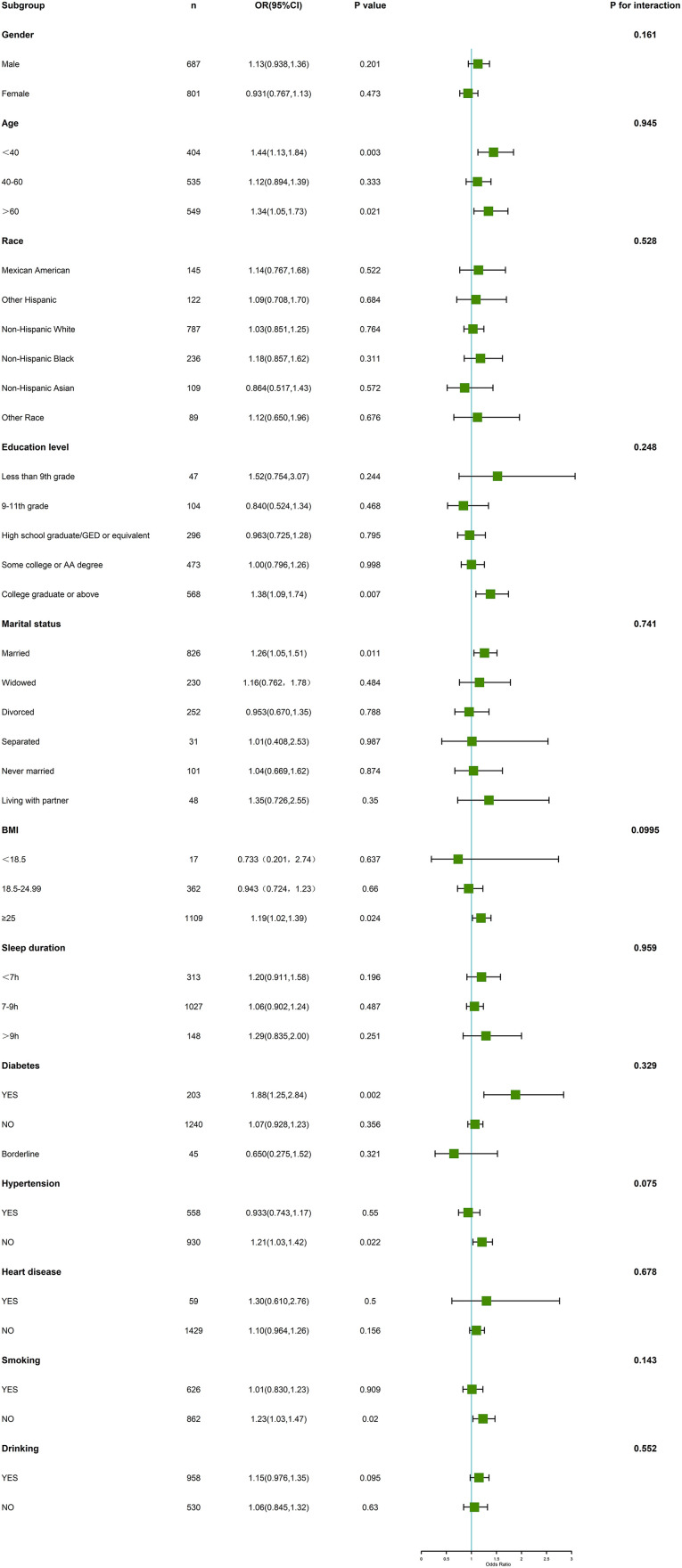
Forest plot of subgroup analysis for PA.

According to subgroup analysis results, the relationship between PA and anxiety symptoms showed significant differences across groups. Specifically, the relationship between PA and anxiety symptoms differed significantly among younger (< 40 years, OR = 1.44, 95% CI: 1.13–1.84, *p* = 0.003) and older adults (> 60 years, OR = 1.34, 95% CI: 1.05–1.73, *p* = 0.021), as well as among individuals with a BMI ≥ 25 (OR = 1.19, 95% CI: 1.02–1.39, *p* = 0.024); the association between PA and anxiety symptoms was stronger in these groups. Additionally, individuals with higher education (college graduates or above, OR = 1.38, 95% CI: 1.09–1.74, *p* = 0.007), married individuals (OR = 1.26, 95% CI: 1.05–1.51, *p* = 0.011), and diabetic individuals (OR = 1.88, 95% CI: 1.25–2.84, *p* = 0.002) exhibited a stronger association between PA and anxiety symptoms than other individuals. Further analyses revealed that individuals without hypertension (OR = 1.21, 95% CI: 1.03–1.42, *p* = 0.022) and non-smokers (OR = 1.23, 95% CI: 1.03–1.47, *p* = 0.02) exhibited a stronger negative association between PA and anxiety symptoms than other individuals. However, in certain groups, the association between PA and anxiety symptoms did not reach statistical significance. For example, gender (male OR = 1.13, 95% CI: 0.938–1.36, *p* = 0.201; female OR = 0.931, 95% CI: 0.767–1.13, *p* = 0.473), race (non-Hispanic white OR = 1.03, 95% CI: 0.851–1.25, *p* = 0.764), and sleep duration (< 7 hours OR = 1.20, 95% CI: 0.911–1.58, *p* = 0.196; 7–9 hours OR = 1.06, 95% CI: 0.902–1.24, *p* = 0.487; > 9 hours OR = 1.29, 95% CI: 0.835–2.00, *p* = 0.251) were factors for which PA did not show a significant association with anxiety symptoms. Detailed information about the association between PA and anxiety symptoms is displayed in Fig 3.

#### Association between PHR and anxiety symptoms.

**Fig 4:**
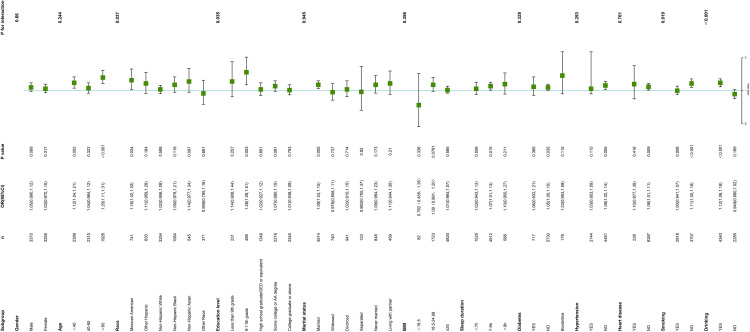
Forest plot of subgroup analysis for PHR.

Similar to PA’s association with anxiety symptoms, the association between PHR and anxiety symptoms also revealed significant differences across groups. Specifically, the association between PHR and anxiety symptoms was stronger among individuals aged < 40 years (OR = 1.12, 95% CI: 1.04–1.21, *p* = 0.002) and ≥ 60 years (OR = 1.20, 95% CI: 1.11–1.31, *p* < 0.001). Additionally, there was a similarly significant association between PHR and anxiety symptoms among Mexican Americans (OR = 1.16, 95% CI: 1.02–1.33, *p* = 0.024), individuals with a 9th-11th grade education level (OR = 1.28, 95% CI: 1.09–1.51, *p* = 0.003), and married individuals (OR = 1.09, 95% CI: 1.03–1.15, *p* = 0.005). Moreover, lifestyle factors also moderated this relationship. Specifically, PHR exerted more significant effects on anxiety among individuals with sleep duration of 7−9 hours (OR = 1.07, 95% CI: 1.01–1.13, *p* = 0.015), non-smokers (OR = 1.11, 95% CI: 1.05–1.18, *p* < 0.001), and individuals with alcohol consumption (OR = 1.12, 95% CI: 1.06–1.18, *p* < 0.001). Additionally, the effect of PHR on anxiety was more significant in chronic disease patients without diabetes (OR = 1.05, 95% CI: 1.00–1.10, *p* = 0.035), without hypertension (OR = 1.08, 95% CI: 1.02–1.14, *p* = 0.005), and without heart disease (OR = 1.06, 95% CI: 1.01–1.11, *p* = 0.009); the association between PHR and anxiety disorders was similarly significant in these groups. However, similar to PA analysis results, the association between PHR and anxiety symptoms did not show statistically significant differences in certain groups. For example, some factors (such as gender and BMI) did not reveal significant differences in the relationship between PHR and anxiety disorders. Although some association was observed in certain subgroups, most results did not reach statistical significance. Detailed information about the association between PA and anxiety symptoms is displayed in Fig 4.

### Interactive analysis

#### PA interactivity analysis.

PA interactivity analysis results indicated that the vast majority of variables did not significantly interact (*p* > 0.05) with the relationship between PA and anxiety symptoms. Specifically, gender (*p* = 0.161), age (*p* = 0.945), race (*p* = 0.528), education level (*p* = 0.248), marital status (*p* = 0.741), BMI (*p* = 0.0995), sleep duration (*p* = 0.959), and chronic diseases (diabetes: *p* = 0.329; hypertension: *p* = 0.075; heart disease: *p* = 0.678) did not significantly modulate the relationship between PA and anxiety symptoms. This suggested that the effect of PA on alleviating anxiety symptoms demonstrated a high degree of consistency across groups. Although factors such as BMI and hypertension approached significance in the interaction (*p* < 0.1), these differences did not reach statistical significance (*p* > 0.05), further supporting the generalized role of PA across individuals.

#### PHR interactivity analysis.

Similar to PA interactivity analysis results, the association between PHR and anxiety symptoms showed significant differences across groups. Specifically, the association between PHR and anxiety symptoms was stronger among individuals aged < 40 years (OR = 1.12, 95% CI: 1.04–1.21, *p* = 0.002) and ≥ 60 years (OR = 1.20, 95% CI: 1.11–1.31, *p* < 0.001) than among other individuals. Additionally, Mexican Americans (OR = 1.16, 95% CI: 1.02–1.33, *p* = 0.024), individuals with a 9th-11th grade education level (OR = 1.28, 95% CI: 1.09–1.51, *p* = 0.003), and married individuals (OR = 1.09, 95% CI: 1.03–1.15, *p* = 0.005) demonstrated a similarly significant association between PHR and anxiety symptoms. Moreover, lifestyle factors also moderated this relationship. Specifically, sleep duration of 7−9 hours (OR = 1.07, 95% CI: 1.01–1.13, *p* = 0.015), non-smokers (OR = 1.11, 95% CI: 1.05–1.18, *p* < 0.001), and alcohol consumption (OR = 1.12, 95% CI: 1.06–1.18, *p* < 0.001) were associated with a more significant effect of PHR on anxiety. Additionally, the effect of PHR on anxiety was more significant in chronic disease patients without diabetes (OR = 1.05, 95% CI: 1.00–1.10, *p* = 0.035), without hypertension (OR = 1.08, 95% CI: 1.02–1.14, *p* = 0.005), and without heart disease (OR = 1.06, 95% CI: 1.01–1.11, *p* = 0.009). The association between PHR and anxiety disorders was similarly significant in these groups. However, similar to PA analysis results, the association between PHR and anxiety symptoms did not show statistically significant differences in certain groups. For example, some factors (such as gender and BMI) did not show significant differences in the relationship between PHR and anxiety disorders. Although some associations were observed in certain subgroups, most results did not reach statistical significance. Detailed information is shown in Fig 4.

In the analysis of the relationship between PHR and anxiety symptoms, most variables did not significantly affect their association (*p* > 0.05). Gender (*p* = 0.66), age (*p* = 0.244), marital status (*p* = 0.945), BMI (*p* = 0.356), sleep duration (*p* = 0.193), and chronic diseases (e.g., diabetes *p* = 0.329; hypertension *p* = 0.293; and heart disease *p* = 0.761) did not exhibit significant moderating effects on the relationship between PHR and anxiety symptoms. However, race (*p* = 0.037), education level (*p* = 0.035), smoking (*p* = 0.019), and alcohol consumption (*p* < 0.001) moderated the relationship between PHR and anxiety symptoms, demonstrating a significant interaction (*p* < 0.05).

## Discussion

This study systematically examined the relationship between PA, PHR, and the risk of anxiety disorders using NHANES 2015–2023 data. Logistic regression analyses, unrestricted cubic splines, and subgroup analyses were used to validate the robust associations between these variables and further reveal their potential heterogeneity.

Logistic regression analysis results indicated that PA was significantly associated with a reduced risk of anxiety disorders (Model 3, p = 0.007), supporting that PA could positively affect mental health. Consistently, regular PA has been shown to effectively reduce anxiety symptoms by modulating neurobiological mechanisms and alleviating psychological distress [23]. Specifically, regarding the exercise intensity, this study found that high-intensity PA was significantly more effective in alleviating anxiety disorders than mild-intensity PA, which did not exhibit a significant effect. These results highlight the crucial role of exercise intensity in alleviating anxiety disorders. High-intensity exercise may offer more substantial benefits through enhanced physiological responses and psychological improvements. The finding of this article aligns with existing literature. For example, Jens Plag has randomized 33 patients with Generalized Anxiety Disorder (GAD) to 12 days of either high-intensity interval training (HIIT) or low-intensity training (LIT), demonstrating that HIIT typically has approximately twice the impact of LIT and is highly effective and fast-acting in GAD [24]. Moreover, a previous study has shown that high-intensity exercise notably alleviates anxiety symptoms by activating the nervous system and promoting neurotransmitter balance [25]. However, Emily M. Paolucci has suggested that moderate-intensity exercise may be the optimal intensity for promoting mental health by lowering tumor necrosis factor (TNF)-α [26]. This finding somewhat contrasts with the results of this study. The potential reasons for this discrepancy include variations in intervention design, individual adaptations, and psychological status.

Subgroup analyses revealed significant differences in the relationship between PA and the risk of anxiety disorders across different groups. Specifically, the relationship between PA and anxiety symptoms was more pronounced among younger (< 40 years) and older (> 60 years) age groups. Younger individuals experienced more significant psychological benefits from PA, especially in reducing academic or occupational stress-induced anxiety. A previous study has also demonstrated that PA positively affects both physical and psychological health of the elderly population, particularly those facing physical deterioration and social isolation [27].

Among individuals with a BMI ≥ 25, this study similarly found a negative association between PA and anxiety symptoms. Consistent results have been reported in previous studies. For example, Zhao has suggested that a higher BMI is typically associated with a greater psychological burden [28]. On the other hand, Lan Chen’s study has indicated that PA can alleviate anxiety by reducing the burden on the body [29]. Furthermore, the present study revealed that negative associations between PA and anxiety disorders were also more pronounced in individuals with higher education level, married individuals, and diabetic individuals, further validating the positive effects of PA in specific groups. Additionally, individuals without hypertension and non-smokers exhibited stronger negative associations between PA and anxiety disorders, suggesting that effective blood pressure control and smoking cessation may enhance the mental health benefits of PA. In support of these findings, studies have indicated that chronic conditions, such as hypertension and smoking, could increase psychological burden [30,31], and PA can alleviate these burdens.

However, the relationship between PA and anxiety disorders did not reach significance in some groups, particularly with regrading to gender, race, and sleep duration. Specifically, the lack of significance in gender differences may reflect variations between males and females in exercise adaptations and performance in response to anxiety disorders. The lack of significance for the race factor may be related to variations in cultural background, lifestyle, or social support systems, and further exploration is warranted through cross-cultural studies in the future. Regarding sleep duration, although a meta-analysis by Alexander J. Scott of 65 studies has shown that improved sleep quality leads to greater improvements in mental health [32], this study did not reveal a significant role of sleep duration in the relationship between PA and anxiety disorders. Possible reasons for this include the greater influence of sleep quality and regularity on the relationship between PA and anxiety disorders, as well as the insufficient control for these factors in this study, which may result in the lack of significant effects of sleep duration. Therefore, future research should further explore the potential moderating effects of factors such as sleep quality and regularity on the relationship between PA and anxiety disorders.

This study is the first to investigate the relationship between the PHR and the risk of anxiety disorders, revealing that a higher PHR was significantly associated with a lower risk of anxiety disorders (Model 3, *p* = 0.002). This finding supports the protective role of the anti-inflammatory and antioxidant effects of HDL-C in mental health and suggests that platelet activity may play a significant role in the development of anxiety disorders [33]. A study by Mario Gennaro Mazza has suggested that platelet overactivation may exacerbate the psychological stress response, thereby increasing the risk of anxiety disorders [34]. By combining HDL-C with platelet activity as PHR, this study validated the potential clinical value of this biomarker in assessing the risk of anxiety disorders and offers a new perspective on mental health risk assessment.

Unrestricted cubic spline analysis revealed a significant non-linear relationship between PHR and the risk of anxiety disorders. Low PHR values may lead to an enhanced inflammatory response due to platelet overactivation [35]. On the other hand, excessive PHR values may disrupt inflammatory regulatory mechanisms due to abnormally elevated HDL-C levels [36]. The present study revealed that moderate PHR values (approximately 4.51 to 10.18) may offer stronger protection against anxiety disorders. Consistently, a previous study has suggested that the biological mechanisms of anxiety disorders are complex and involve the interaction of multiple biomarkers [37]. Future research should focus on understanding the differential impact of various PHR levels on anxiety disorders and developing personalized mental health intervention strategies by modulating PHR levels.

The relationship between PHR and anxiety disorders demonstrated significant heterogeneity across groups. Specifically, age was identified as a key factor; the relationship between PHR and anxiety disorders was particularly pronounced among younger (< 40 years) and older (≥ 60 years) age groups. Consistently, Ruthmann F has indicated that anxiety and depression may be associated with differences in cognitive and endothelial functioning in non-clinical younger populations [38]. Younger individuals may alleviate academic and workplace stress-induced anxiety by maintaining better vascular function and immune responses. In older adults, who experience body function decline and social isolation, the role of PHR is particularly crucial, and moderate PHR levels may help mitigate aging-associated physical and psychological stresses [39]. Lifestyle factors also play a significant role in the relationship between PHR and anxiety disorders. Adequate sleep and non-smoking may strengthen the negative association between PHR and anxiety disorders. Similarly, Dionne Morgan’s research has also suggested that sleep improves endocrine and immune system functioning [40], which is consistent with the findings of the present study. In contrast, smoking and excessive alcohol consumption may reduce the protective effect of PHR and exacerbate anxiety symptoms. Our study results suggested that a healthy lifestyle enhances the mitigating effect of PHR on anxiety, whereas an unhealthy lifestyle may diminish this effect.

Additionally, our findings suggested that the socioeconomic background and health status of individuals influenced the relationship between PHR and anxiety disorders. In short, individuals with low socioeconomic status generally experience greater psychological burdens, and changes in PHR have a more pronounced impact on their mental health. The relationship between PHR and anxiety disorders was particularly pronounced among individuals with chronic conditions, especially in those without diabetes or hypertension. Individuals with chronic conditions are often burdened by health management, and appropriate levels of PHR may improve physical health and alleviate psychological stress, thus reducing the risk of anxiety disorders. Consistently, it has been shown that PHR positively affects mental health by modulating physiological status in these groups [19]. Despite the significant relationship between PHR and anxiety disorders in most groups, the role of some factors (such as gender and BMI) did not achieve statistical significance. Gender differences may be attributed to differences in physiological responses and psychological adaptations between males and females. The effect of BMI on the relationship between PHR and anxiety disorders appeared to be more complex and lacked consistency, suggesting that BMI may not be the sole factor influencing the relationship between PHR and anxiety disorders, and the underlying mechanism may vary based on individual differences.

The effects of PA on anxiety disorders are primarily mediated by the modulation of various biological mechanisms. First, mild PA could remarkably improve neurotransmitter homeostasis, particularly serotonin (5-hydroxytryptamine), norepinephrine, and gamma-aminobutyric acid (GABA) [41]. These neurotransmitters play a crucial role in mood regulation, and exercise helps stabilize mood and reduce anxiety symptoms by enhancing the production of these neurotransmitters. Exercise also enhances the activity of GABA, an inhibitory neurotransmitter that prevents the overexcitation of neural signals in the brain, thereby exerting a calming effect and reducing anxiety [42].

Second, PA regulates the function of the hypothalamic-pituitary-adrenal (HPA) axis [43]. Exercise helps lower cortisol levels, a hormone closely associated with stress and anxiety [44]. Moreover, by reducing cortisol levels, exercise diminishes the body’s physiological response to stress, thereby alleviating anxiety symptoms. Additionally, exercise increases brain-derived neurotrophic factor (BDNF) levels [45]. BDNF promotes neuroplasticity, especially in brain regions closely linked to emotion regulation, such as the prefrontal cortex and hippocampus, thereby alleviating anxiety [46].

In addition to modulating neurotransmitters and the HPA axis, PA’s role in anxiety is closely linked to its anti-inflammatory effects. Chronic low-grade inflammation is considered a key factor in the onset and progression of anxiety disorders, and aerobic exercise has been shown to effectively reduce inflammation levels in the body [47]. Furthermore, it has been shown that exercise reduces inflammatory markers, such as C-reactive protein (CRP), interleukin-6 (IL-6), and TNF-α, thus reducing chronic inflammation-triggered anxiety symptoms [48]. Additionally, exercise promotes the secretion of anti-inflammatory factors, such as IL-10, which further alleviates inflammation-induced anxiety [49].

Concurrently, PHR significantly affected the risk of anxiety disorders, with the underlying mechanism involving complex biological pathways. A previous study has revealed that thrombocytosis is strongly associated with increased immune response and inflammation levels [50]. Platelets not only play a crucial role in blood coagulation but also release various biologically active substances, including platelet-derived growth factor (PDGF) and TNF-α, which amplify the inflammatory response by activating immune cells [51,52]. Chronic inflammation has been shown to play a central role in the pathogenesis of various psychiatric disorders, particularly in the development of depression and anxiety, with inflammatory mediators (such as IL-6 and TNF-α) playing a crucial role in their onset and progression [53,54]. By affecting immune cells in the central nervous system, these factors may result in neurotransmitter imbalances, which in turn may trigger anxiety.

In contrast, HDL-C is thought to possess anti-inflammatory and antioxidant properties, which could reverse lipid deposition in blood vessels and enhance vascular endothelial function, thereby alleviating chronic inflammation-associated anxiety symptoms [55]. It has been evidenced that HDL-C improves vascular health by lowering inflammatory markers such as CRP, which consequently helps alleviate anxiety [56]. Moderate PHR values may reduce the risk of anxiety by regulating the balance between platelets and HDL-C, thereby modulating the immune system and inflammatory responses. However, when PHR values are high, platelet activity is excessively enhanced, which may inhibit the protective effects of HDL-C. Overactive platelets are associated with chronic inflammation, further exacerbating anxiety symptoms by releasing factors such as angiotensin II and IL-1β, which amplify inflammatory responses in the nervous system [57]. These excessive inflammatory responses may damage the vascular endothelium and cause vascular dysfunction, which in turn aggravates psychological stress and promotes anxiety. On the other hand, when PHR values are too low, the anti-inflammatory effects of HDL-C may not be fully utilized to effectively inhibit the persistence of low-grade chronic inflammation. This inflammation continues to affect the brain, particularly the regions involved in emotion regulation, such as the prefrontal cortex and amygdala, further exacerbating anxiety symptoms [58]. Additionally, a previous study has revealed that the AMPK (5’AMP-activated protein kinase) pathway plays a critical role in this process [59]. AMPK not only regulates cellular energy homeostasis but also plays a crucial role in modulating the inflammatory response [60]. Activation of the AMPK pathway has been shown to reduce inflammatory responses and improve vascular endothelial function, thereby reducing anxiety symptoms [61].

Therefore, PA and PHR are interconnected in their effects on anxiety disorders. Exercise not only directly modulates neurotransmitters and the HPA axis but also indirectly influences PHR by improving lipid levels and reducing inflammatory responses. A previous study has shown that regular PA improves lipid metabolism, especially by increasing HDL-C levels, which in turn influences platelet activity and reduces the risk of anxiety disorders [62]. Meanwhile, PA further reduces the inflammatory response and anxiety symptoms triggered by PHR by modulating the AMPK pathway [63].

In summary, the interaction between PA and PHR may influence the risk of anxiety disorders by co-regulating immune system function, inflammatory response, and metabolic processes. PA comprehensively reduces anxiety symptoms by improving neurotransmitter balance, modulating the HPA axis, decreasing inflammatory markers, increasing levels of anti-inflammatory factors, and modulating PHR. Therefore, future studies should continue to explore the specific mechanisms of PHR modulation through PA, especially how to optimize PHR through exercise, thereby providing new perspectives and strategies for intervention in anxiety disorders.

### Strengths and limitations

The strengths of this study are primarily reflected in several aspects. First, this study involves a large sample size, and the data are highly representative and comprehensive. A large sample size enhances the statistical validity of the findings and reduces the risk of bias caused by insufficient sample size. Second, this study used various analytical methods (including logistic regression, subgroup analysis, and unrestricted cubic spline analysis) to comprehensively explore the relationship between PA, PHR, and anxiety disorders, thereby verifying the robustness and reliability of the results. Additionally, the data in this study demonstrated high validity, and strict data cleaning and quality control procedures ensured the accuracy of our findings.

Nevertheless, several limitations should be noted in this study. First, as a cross-sectional study, this study cannot explore causal relationships, and only associations between variables can be revealed. Long-term causal relationships must be further validated through longitudinal studies. Second, although extensive cohort data were included, certain factors (e.g., individuals’ specific lifestyles, timeliness of emotional responses, etc.) may not have been fully controlled due to limitations in data sources and study design, and potential confounders cannot be ruled out. Additionally, despite the use of various statistical methods, bias resulting from the choice of analytical approach could not be completely ruled out. The longitudinal design should be enhanced and more granular data are needed in future research to more accurately reveal the causal relationship between PA, PHR, and anxiety disorders.

### Clinical implications and public health implications

This study provides new perspectives on the management of anxiety disorders. First, the findings of this article highlight the potential of PA as an effective intervention for alleviating anxiety symptoms, consistent with recommendations in multiple clinical guidelines. For example, both the American Psychological Association (APA) and the WHO recommend improving mental health through increasing PA, which is particularly effective in alleviating anxiety and depression symptoms. Additionally, the exploration of PHR in this study introduces new potential biomarkers for anxiety disorders, which may contribute to more accurate clinical screening and intervention for anxiety.

From a public health perspective, this study emphasizes lifestyle interventions, especially by increasing PA to reduce anxiety disorder risk. This aligns with existing public health initiatives, with many national and regional guidelines emphasizing the need to increase the prevalence of PA to improve mental health and reduce the burden of mental disorders, including anxiety and depression. Therefore, future public health policies should further promote PA as an effective means of preventing and intervening in anxiety disorders, while focusing on relevant biomarkers (such as PHR) to more accurately assess individuals’ risk of anxiety disorders and develop personalized interventions.

### Future research directions

Future studies should employ a longitudinal design to further validate the causal relationship between PA and PHR in anxiety disorders and investigate their interaction. Additionally, in-depth studies are required to examine the association between PHR and other mental health issues, as well as to explore its underlying mechanisms and identify the optimal intervention strategies for different populations. Studies integrating biological mechanisms will elucidate the central role of PA and PHR in mental health, providing a scientific basis for the development of more precise mental health interventions.

## Conclusion

Overall, the present study demonstrated that both PA and PHR were significantly associated with anxiety symptoms, but the relationship between the two exhibited different patterns across groups with varying demographic characteristics. The effect of PA on anxiety symptoms was relatively generalizable, whereas the relationship between PHR and anxiety symptoms was influenced by race and education level. These findings provide information for developing mental health intervention strategies tailored to different groups, highlighting the potential of PA as an effective reliever of anxiety symptoms and emphasizing the importance of considering demographic differences when evaluating health risk factors.
